# *In Vitro* Activity of Two Cefepime-Based Novel Combinations, Cefepime/Taniborbactam and Cefepime/Zidebactam, against Carbapenemase-Expressing *Enterobacterales* Collected in India

**DOI:** 10.1128/spectrum.04925-22

**Published:** 2023-02-27

**Authors:** Yamuna Devi Bakthavatchalam, Divyaa Elangovan, Subha Vajjiravelu Jaganathan, Nivedhana Subburaju, Abirami Shankar, Yuvasri Manokaran, Sudarsana J., Rema Devi, Sujata Baveja, Sheela Devi, Jayakumar S., Sanjay Bhattacharya, Rudresh S. M., Bineshlal Yesudhason, Vignesh Shetty, Ankur Mutreja, Abi Manesh, George M. Varghese, Charis A. Marwick, Benjamin J. Parcell, Ian H. Gilbert, Balaji Veeraraghavan

**Affiliations:** a Department of Clinical Microbiology, Christian Medical College and Hospital, Vellore, India; b Department of Microbiology, Panimalar Medical College Hospital and Research Institute, Chennai, India; c Department of Microbiology, Meenakshi Medical College and Research Institute, Kanchipuram, India; d Department of Microbiology, Rainbow Children’s Hospital and Perinatal Care, Hyderabad, India; e Department of Microbiology, Baby Memorial Hospital, Kozhikode, India; f Department of Microbiology, Dr. Somervell Memorial CSI Medical College and Hospital, Thiruvananthapuram, India; g Department of Microbiology, Lokmanya Tilak Municipal General Hospital and Medical College (Sion Hospital), Mumbai, India; h Department of Microbiology, Pondicherry Institute of Medical Sciences, Kalapet, India; i Department of Microbiology, Saveetha Medical College and Hospital, Chennai, India; j Department of Microbiology, Tata Medical Center, Kolkata, India; k Department of Microbiology, ESI Post Graduate Institute of Medical Science and Research, Bengaluru, India; l Department of Medicine, Cambridge Institute of Therapeutic Immunology & Infectious Disease (CITIID), University of Cambridge, Cambridge, United Kingdom; m Department of Infectious Disease, Christian Medical College and Hospital, Vellore, India; n Population Health and Genomics, University of Dundee, Dundee, United Kingdom; o Ninewells Hospital and Medical School, Dundee, United Kingdom; p Division of Biological Chemistry and Drug Discovery, University of Dundee, Dundee, United Kingdom; Veterans Affairs Northeast Ohio Healthcare System

**Keywords:** cefepime, taniborbactam, zidebactam, carbapenemases, *Enterobacterales*, β-lactam enhancer, β-lactamase inhibitor

## Abstract

In recent times, discovery efforts for novel antibiotics have mostly targeted carbapenemase-producing Gram-negative organisms. Two different combination approaches are pertinent: β-lactam–β-lactamase inhibitor (BL/BLI) or β-lactam–β-lactam enhancer (BL/BLE). Cefepime combined with a BLI, taniborbactam, or with a BLE, zidebactam, has been shown to be promising. In this study, we determined the *in vitro* activity of both these agents along with comparators against multicentric carbapenemase-producing *Enterobacterales* (CPE). Nonduplicate CPE isolates of Escherichia coli (*n* = 270) and Klebsiella pneumoniae (*n* = 300), collected from nine different tertiary-care hospitals across India during 2019 to 2021, were included in the study. Carbapenemases in these isolates were detected by PCR. E. coli isolates were also screened for the presence of the 4-amino-acid insert in penicillin binding protein 3 (PBP3). MICs were determined by reference broth microdilution. Higher MICs of cefepime/taniborbactam (>8 mg/L) were linked to NDM, both in K. pneumoniae and in E. coli. In particular, such higher MICs were observed in 88 to 90% of E. coli isolates producing NDM and OXA-48-like or NDM alone. On the other hand, OXA-48-like-producing E. coli or K. pneumoniae isolates were nearly 100% susceptible to cefepime/taniborbactam. Regardless of the carbapenemase types and the pathogens, cefepime/zidebactam showed potent activity (>99% inhibited at ≤8 mg/L). It seems that the 4-amino-acid insert in PBP3 (present universally in the study E. coli isolates) along with NDM adversely impact the activity of cefepime/taniborbactam. Thus, the limitations of the BL/BLI approach in tackling the complex interplay of enzymatic and nonenzymatic resistance mechanisms were better revealed in whole-cell studies where the activity observed was a net effect of β-lactamase inhibition, cellular uptake, and target affinity of the combination.

**IMPORTANCE** The study revealed the differential ability of cefepime/taniborbactam and cefepime/zidebactam in tackling carbapenemase-producing Indian clinical isolates that also harbored additional mechanisms of resistance. NDM-expressing E. coli with 4-amino-acid insert in PBP3 are predominately resistant to cefepime/taniborbactam, while the β-lactam enhancer mechanism-based cefepime/zidebactam showed consistent activity against single- or dual-carbapenemase-producing isolates including E. coli with PBP3 inserts.

## INTRODUCTION

Resistance to carbapenems in *Enterobacterales* is principally mediated by carbapenemases, which are either serine-β-lactamases (SBLs; class A, C, and D) or metallo-β-lactamases (MBLs; class B). Klebsiella pneumoniae carbapenemase (KPC) and oxacillinase-48-like (OXA-48-like) are serine-β-lactamases while New Delhi metallo-β-lactamase, imipenemase (IMP), and Verona integron-encoded MBLs (VIMs) are metallo-β-lactamases (1). The genes encoding these β-lactamases exist in plasmids and integrons and have been disseminated globally. However, the epidemiology of each of these carbapenemases among *Enterobacterales* is heterogeneous. NDMs are endemic in India and China, predominately in Escherichia coli and also in K. pneumoniae (2, 3), albeit many clinical cases involving NDM-*Enterobacterales* are regularly reported from other parts of the world such as South Korea, Iran, and Italy (4–6). OXA-48-like enzymes are predominant in Europe, northern Africa, the Middle East, and increasingly in India (7, 8). KPCs are the most frequent carbapenemases in the United States, Greece, Italy, and China while rarely documented in India (9, 10). From the antibiotic resistance perspective, India is a hot spot with carbapenem resistance rates consistently exceeding 30% in *Enterobacterales* (10), and therefore, Indian isolates reflect the current as well as future challenges of antibiotic resistance which other parts of the world may face eventually.

Among the licensed novel β-lactamase inhibitors, avibactam and relebactam are based on the diazabicyclooctane (DBO) pharmacophore; avibactam inhibits Ambler class A, C, and some D enzymes and relebactam inhibits class A and C enzymes, while both lack MBL-inhibitory activity (11). Vaborbactam, a boronate-based inhibitor with an inhibitory spectrum akin to that of relebactam, also lacks MBL inhibition (12); however, the structural changes in this pharmacophore have led to newer boronates with an extended β-lactamase-inhibitory spectrum (13). In this series of β-lactamase inhibitors, taniborbactam is shown to inhibit all four Ambler class A, B, C, and D enzymes (except IMP), and its combination with cefepime has recently completed a registrational phase 3 trial for the indication of complicated urinary tract infection (cUTI) or acute pyelonephritis (AP) in adults (14, 15).

Chemical modifications of DBOs led to the identification of a novel bicyclo-acyl hydrazide (BCH) pharmacophore, derivatives of which have shown a selective pan-Gram-negative penicillin binding protein 2 (PBP2) binding feature, as well as inhibiting class A and C β-lactamases. Zidebactam is the first-in-class BCH being combined with PBP3-targeting cefepime (16–18). The antibacterial activity of this combination is mediated by synergistic PBP inactivation, in addition to the β-lactamase inhibition feature of zidebactam. This novel mode of action endowed the combination with broad-spectrum activity against Gram-negative organisms expressing all four Ambler class β-lactamases (19–23). Cefepime/zidebactam is in phase 3 clinical development for the indication of cUTI or AP treatment (ClinicalTrials.gov identifier NCT04979806).

In view of these two cefepime-based novel combinations utilizing divergent mechanisms of action in overcoming carbapenemases, we sought to determine their *in vitro* activity against a collection of contemporary, genetically confirmed carbapenemase-producing *Enterobacterales* isolates collected from different hospitals in India.

## RESULTS

The MIC distributions for cefepime/taniborbactam, cefepime/zidebactam, and other comparators are shown in Table 1 (E. coli) and Table 2 (K. pneumoniae). The distributions are provided for both the organisms under three subsets: NDM, NDM plus OXA-48-like, and OXA-48-like producers.

**TABLE 1:** MIC distribution for E. coli categorized by carbapenemase type^*a*^

Antibiotic [carbapenemase(s) produced (no. of isolates)]	No. of isolates with MIC (mg/L)												% S	MIC (mg/L)	
	≤0.06	0.12	0.25	0.5	1	2	4	8	16	32	64	>64		MIC_50_	MIC_90_
NDM producers (*n* = 211)															
Cefepime											4	207	0	>64	>64
Imipenem						1		8	48	89	46	19	0	32	64
Meropenem							3	2	7	20	57	122	0	64	>64
Ceftazidime/avibactam									1	6	10	194	0	>64	>64
Imipenem/relebactam						1	2	9	58	82	44	15	0	32	64
Meropenem/vaborbactam							2	3	6	26	56	118	0.94	>64	>64
Cefepime/taniborbactam				3	7		1	15	31	63	57	34	12.3	32	>64
Cefepime/zidebactam	53	85	44	23	5	1							100	0.12	0.5
Aztreonam/avibactam	14	1	9	12	31	37	39	40	26	2			67.8	4	16
NDM plus OXA-48-like producers (*n* = 39)															
Cefepime											2	37	0	>64	>64
Imipenem									10	11	13	5	0	32	>64
Meropenem										3	10	26	0	64	>64
Ceftazidime/avibactam									1	1	1	36	0	>64	>64
Imipenem/relebactam								2	7	16	9	5	0	32	>64
Meropenem/vaborbactam										3	9	27	0	>64	>64
Cefepime/taniborbactam					2		1	1	9	13	7	6	10.3	32	>64
Cefepime/zidebactam	8	15	11	2	1	1	1						100	0.12	0.5
Aztreonam/avibactam	5	1	1	3	4	3	9	8	2	1	1	1	66.67	4	16
OXA-48-like producers (*n* = 20)															
Cefepime	1				1		1	4	3	1		9	35	16	>64
Imipenem	1	1	2	4	5	5	1	1					65	1	2
Meropenem	2	3	1	8	4		1	1					90	0.5	1
Ceftazidime/avibactam	3	1	1		6	6	2	1					100	1	4
Imipenem/relebactam	2	2	6	2	3	4	1						75	0.25	2
Meropenem/vaborbactam	5	2	2	6	3	1		1					95	0.5	1
Cefepime/taniborbactam	1			3	6	5	3	1			1		95	1	4
Cefepime/zidebactam	5	7	4	1	2		1						100	0.12	1
Aztreonam/avibactam	4		2	1		1	4	1	6	1			60	4	16

a The shaded boxes indicate the nonsusceptible range per CLSI criteria. % S, percent susceptibility was evaluated based on CLSI interpretive criteria published in M100, 32nd edition (43). Cefepime/zidebactam MICs were determined using a 1:1 ratio. For all the β-lactam–β-lactamase-inhibitor-based combinations, a fixed inhibitor concentration of 4 mg/L was employed, except for vaborbactam, for which a fixed 8-mg/L concentration was employed. For cefepime-based combinations, the percent susceptibilities were determined based on the susceptible–dose-dependent breakpoint of ≤8 mg/L for *Enterobacterales*. For aztreonam/avibactam, the percent susceptibilities were determined based on aztreonam CLSI interpretive criteria for *Enterobacterales*.

**TABLE 2:** MIC distribution for K. pneumoniae categorized by carbapenemase type^*a*^

Antibiotic [carbapenemase(s) produced (no. of isolates)]	No. of isolates with MIC (mg/L)												% S	MIC (mg/L)	
	≤0.06	0.12	0.25	0.5	1	2	4	8	16	32	64	>64		MIC_50_	MIC_90_
NDM producers (*n* = 47)															
Cefepime									2	4	8	33	0	>64	>64
Imipenem								3	7	17	9	11	0	32	>64
Meropenem							1		4	10	9	23	0	64	>64
Ceftazidime/avibactam												47	0	>64	>64
Imipenem/relebactam							1	6	5	17	8	10	0	32	>64
Meropenem/vaborbactam							1		3	2	19	22	2.1	64	>64
Cefepime/taniborbactam	1	1	2	8	12	3	7	1	3	5	3	1	74.5	2	32
Cefepime/zidebactam	1	5	12	7	8	7	4	3					100	0.5	4
Aztreonam/avibactam	21	17	6	1		2							100	0.12	0.25
NDM plus OXA-48-like producers (*n* = 122)															
Cefepime										2	7	113	0	>64	>64
Imipenem							2	7	4	9	23	77	0	>64	>64
Meropenem								2	2	6	16	96	0	>64	>64
Ceftazidime/avibactam												122	0	>64	>64
Imipenem/relebactam					2	2	5	2	6	11	16	78	1.6	>64	>64
Meropenem/vaborbactam							1	4		9	12	96	0.81	>64	>64
Cefepime/taniborbactam				3	5	4	5	6	19	37	29	14	18.9	32	>64
Cefepime/zidebactam	1	3	9	15	41	21	18	13		1			99.18	1	8
Aztreonam/avibactam	13	36	55	12	3		2		1				99.18	0.25	0.5
OXA-48-like producers (*n* = 131)															
Cefepime					2	1	5	2	2	2	10	107	7.63	>64	>64
Imipenem					6	36	52	24	3	4	2	4	4.6	4	8
Meropenem					3	1	6	22	48	40	8	3	2.3	16	32
Ceftazidime/avibactam		2	14	70	35	8	2						100	0.5	1
Imipenem/relebactam			1	1	26	52	29	10	6	2	2	2	21.37	2	8
Meropenem/vaborbactam					4		5	28	61	21	11	1	6.87	16	32
Cefepime/taniborbactam	5	2	1	10	44	44	20	5					100	4	4
Cefepime/zidebactam	1	3	13	28	66	20							100	1	2
Aztreonam/avibactam	19	47	51	11	2	1							100	0.12	0.5

a The shaded boxes indicate the nonsusceptible range per CLSI criteria. % S, percent susceptibility was evaluated based on CLSI interpretive criteria published in M100, 32nd edition (43). Cefepime/zidebactam MICs were determined using a 1:1 ratio. For all the β-lactam–β-lactamase-inhibitor-based combinations, a fixed inhibitor concentration of 4 mg/L was employed, except for vaborbactam, for which a fixed 8-mg/L concentration was employed. For cefepime-based combinations, the percent susceptibilities were determined based on the susceptible–dose-dependent breakpoint of ≤8 mg/L for *Enterobacterales*. For aztreonam/avibactam, the percent susceptibilities were determined based on the aztreonam interpretive criteria for *Enterobacterales*.

### E. coli.

Against NDM-producing E. coli isolates (*n* = 211), activities of cefepime/taniborbactam and cefepime/zidebactam were quite contrasting. Applying cefepime’s susceptible–dose-dependent (SDD) breakpoint of ≤8 mg/L, the susceptibility to cefepime/taniborbactam was only 12.3% (26 of 211). The modal MIC was 32 mg/L, 2 dilutions higher than cefepime’s SDD breakpoint. On the other hand, cefepime/zidebactam inhibited all the isolates at ≤2 mg/L. Aztreonam/avibactam showed a wide range of MICs with most isolates in the range of 1 to 16 mg/L. Purely based on the potency comparison, aztreonam/avibactam was superior to cefepime/taniborbactam, though both were inferior to cefepime/zidebactam.

E. coli isolates harboring both NDM and OXA-48-like were relatively few (*n* = 39), and the MIC distribution pattern of antibiotics was no different from that for the NDM-alone subset. At ≤8 mg/L, cefepime/taniborbactam inhibited 4/39 (10.3%) isolates, while for cefepime/zidebactam all the isolates were inhibited at ≤4 mg/L. Aztreonam/avibactam showed a scattered MIC distribution. The smallest subset among E. coli was the OXA-48-like producers (*n* = 20); cefepime/taniborbactam had much better activity than against other subsets with MIC values of ≤8 mg/L for 19/20 (95%) isolates. Cefepime/zidebactam continued to show the potency advantage with all the isolates inhibited at ≤4 mg/L, while aztreonam/avibactam again had a wider MIC distribution. To note, a substantial number of OXA-48-producing E. coli isolates were susceptible to carbapenems.

The 4-amino-acid insert in PBP3 was detected in 97% (*n* = 263/270) of E. coli isolates regardless of the type of carbapenemase expressed (NDM and/or OXA-48 like producers, *n* = 243/250; solely OXA-48-like producers, *n* = 20/20).

Table 3 shows the relationship between the MIC distributions of aztreonam/avibactam and cefepime/taniborbactam. Of 89 E. coli isolates with nonsusceptibility to aztreonam/avibactam (MICs of ≥8 mg/L), 79/89 (89%) were also nonsusceptible to cefepime/taniborbactam (MICs of ≥16 mg/L). Even among aztreonam/avibactam-susceptible E. coli isolates (MICs of <8 mg/L), a high proportion of isolates (142 of 181, 79%) were nonsusceptible to cefepime/taniborbactam.

**TABLE 3:** MIC distribution of cefepime/taniborbactam versus aztreonam/avibactam for all E. coli isolates (*n* = 270)^*a*^

Aztreonam/avibactam MIC (mg/L)	No. of isolates inhibited												
	At indicated cefepime/taniborbactam MIC (mg/L)												Total
	≤0.06	0.12	0.25	0.5	1	2	4	8	16	32	64	>64	
≤0.06	1			3	10	1	2		3	1		2	23
0.12					1					1			2
0.25				1		1			3	3	3	1	12
0.5						1			3	7	3	2	16
1								6	10	8	6	5	35
2						1		4	5	19	8	4	41
4				2	2			3	9	11	18	7	52
8					1			2	5	17	12	12	49
16					1	1	3	2	2	7	12	6	34
32											3	1	4
64										1			1
>64										1			1

Total	1	0	0	6	15	5	5	17	40	76	65	40	270

a Of 89 E. coli isolates with nonsusceptibility to aztreonam/avibactam (MICs ≥ 8 mg/L), 79 (88.8%) were also nonsusceptible to cefepime/taniborbactam. Even among aztreonam/avibactam-susceptible E. coli isolates (MICs of ≤8 mg/L), a high proportion of isolates (142 of 181, 79%) were nonsusceptible to cefepime/taniborbactam. For aztreonam/avibactam, CLSI’s aztreonam interpretive criterion is used. For cefepime/taniborbactam, CLSI’s cefepime interpretive criterion is used. Shaded boxes represent isolates with identical MICs of aztreonam/avibactam and cefepime/taniborbactam.

### K. pneumoniae.

Cefepime/taniborbactam showed improved activity against NDM-producing K. pneumoniae with 74.5% inhibition compared to 12.3% inhibition of NDM-producing E. coli isolates at ≤8 mg/L, while at the same cefepime concentration, cefepime/zidebactam inhibited 100% of isolates. Aztreonam/avibactam demonstrated potent (100% inhibition at ≤2 mg/L) activity against NDM-producing K. pneumoniae. Against NDM plus OXA-48-like producers (*n* = 122), cefepime/taniborbactam displayed limited activity with MIC values at ≤8 mg/L for only 23 (18.9%) isolates, while for cefepime/zidebactam all isolates, barring one, were inhibited at ≤8 mg/L. Aztreonam/avibactam was also potent against this dual-carbapenemase subset. Finally, against OXA-48-like-producing K. pneumoniae isolates (*n* = 131), the largest subset in K. pneumoniae, all three combinations, cefepime/taniborbactam, cefepime/zidebactam, and aztreonam/avibactam, were highly potent (100% inhibition at ≤8 mg/L, ≤2 mg/L, and ≤2 mg/L, respectively).

Since zidebactam has been reported to possess significant standalone activity against *Enterobacterales*, we examined whether cefepime/zidebactam is able to show synergistic activity against isolates with elevated zidebactam MICs by determining cefepime/zidebactam MICs for such isolates. These isolates were essentially K. pneumoniae (*n* = 141), of which 99.3% were inhibited at ≤8 mg/L of cefepime/zidebactam while the MICs of standalone zidebactam for all the isolates were ≥32 mg/L (Table 4).

**TABLE 4:** MIC distribution of cefepime/zidebactam for K. pneumoniae (*n* = 141) with elevated MICs for zidebactam (≥32 mg/L)

Cefepime/zidebactam MIC (mg/L)^*a*^	No. of isolates with MIC	% cumulative inhibition
≤0.06	3	2.1
0.12	3	4.3
0.25	11	12.1
0.5	23	28.4
1	44	59.6
2	25	77.3
4	16	88.7
8	15	99.3
16	0	99.3
32	1	100

a Cefepime/zidebactam MICs were determined at a 1:1 ratio. The MIC_50_ was 1 mg/L, and the MIC_90_ was 8 mg/L.

Overall, in the case of both E. coli and K. pneumoniae, other recently approved β-lactam–β-lactamase inhibitors investigated, ceftazidime/avibactam, imipenem/relebactam, and meropenem/vaborbactam, expectedly lacked meaningful activity against subsets producing NDM alone or NDM plus OXA-48-like. Against OXA-48-like producers, ceftazidime/avibactam showed universal inhibition at its susceptibility breakpoint of ≤8 mg/L. Imipenem/relebactam and meropenem/vaborbactam were not active against OXA-48-like-harboring K. pneumoniae, but substantial activity was observed for OXA-48-like-harboring E. coli. However, the latter activities were essentially attributed to the carbapenems alone as both relebactam and vaborbactam are not known to inhibit OXA-48 variants.

## DISCUSSION

Unlike other antibiotic resistance hot spots such as Greece, China, and Italy, India presents a more disturbing trend in terms of high prevalence of dual carbapenemases (NDM plus OXA-48-like) among *Enterobacterales* (7) and therefore needs access to antibiotics that are able to tackle such organisms. Among the late-stage pipeline antibiotics, based on the published data, cefepime/taniborbactam and cefepime/zidebactam seem to hold such promise. The present study was designed to test both these combinations against a challenging panel of carbapenemase-producing isolates collected in India.

Though cefepime is the common β-lactam backbone, the modes of action of cefepime/taniborbactam and cefepime/zidebactam are quite divergent (Table 5). For instance, the activity of cefepime/taniborbactam entirely relies on taniborbactam’s ability to spare cefepime, by inhibiting cefepime-hydrolyzing β-lactamases. On the other hand, in cefepime/zidebactam, the PBP2 binding action of zidebactam plays the pivotal role in overcoming cefepime-impacting resistance mechanisms. By what is termed “β-lactam enhancer” action, cefepime and zidebactam have been reported to concurrently inactivate multiple PBPs, thereby triggering synergistic and pleiotropic bactericidal action which is independent of β-lactamase inhibition (19).

**TABLE 5:**
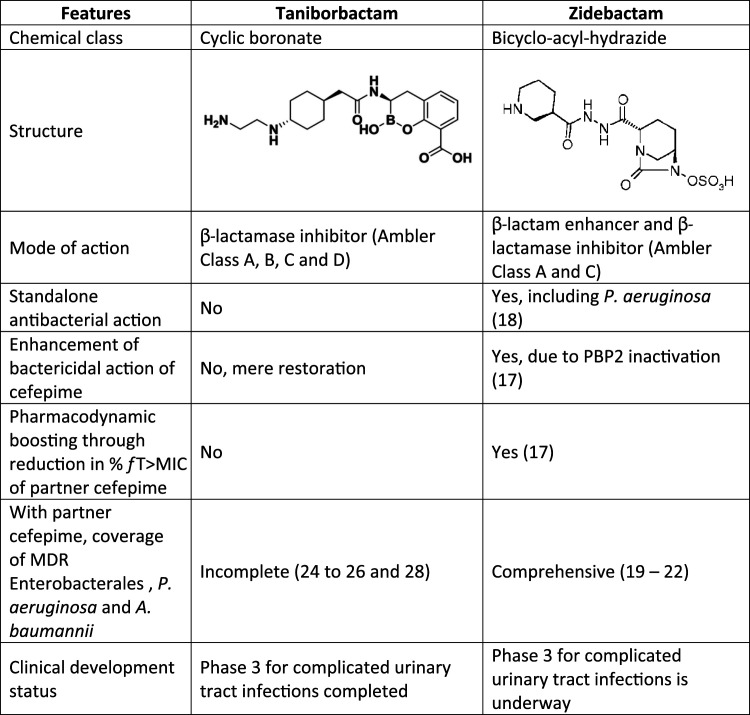
Comparative profile of taniborbactam and zidebactam^*a*^

a For sources of data in the table, please see references 17 to 22 and references 24 to 26 and 28. MDR, multidrug resistant; P. aeruginosa, Pseudomonas aeruginosa; A. baumannii, Acinetobacter baumannii; % *f* T>MIC, proportion of time during which antibiotic concentration remains above MIC.

To facilitate the activity comparison between the antibiotics, E. coli and K. pneumoniae isolates used in this study were categorized into three subsets (NDM, NDM plus OXA-48-like, and OXA-48-like) reflecting the prevailing resistance scenario in high-resistance regions like India and China. Analysis of cefepime/taniborbactam MICs obtained in this study revealed the following activity pattern: good activity (≥95% inhibition at ≤8 mg/L) against isolates expressing OXA-48-like as the only carbapenemase, which is in agreement with previous reports (24–26), and suboptimal activity (<80% inhibition at ≤8 mg/L) for isolates producing NDM alone or NDM plus OXA-48-like. In particular, cefepime/taniborbactam activity was weaker (~10% to 12% inhibition at ≤8 mg/L) against E. coli harboring NDM with or without OXA-48-like. This is striking since cefepime is known to be stable to OXA-48-like β-lactamases (27), and therefore, against such organisms, taniborbactam’s role was limited to tackling the NDM and other coexpressed extended-spectrum β-lactamases (ESBLs).

Even though taniborbactam has been reported to possess potent inhibitory activity (50% inhibitory concentration [IC_50_]) against the purified NDM variants and many ESBLs (28), higher MICs obtained against E. coli producing NDM and OXA-48-like or NDM alone led us to undertake an additional investigation. Based on previous publications (29–31), we analyzed PBP3 for the presence of 4-amino-acid insertions among E. coli isolates and found that an overwhelming proportion of isolates (97%) harbored the inserts regardless of the carbapenemase type. It has been reported previously that those β-lactams that heavily rely on the PBP3 engagement for their antibacterial action are adversely impacted by the presence of amino acid inserts in PBP3 of E. coli (32, 33). Further, it has also been proposed that the presence of amino acid inserts in PBP3 predisposes the organism to acquire a carbapenemase (34). This is evident from the present study as 97% of carbapenemase-producing E. coli isolates harbored PBP3 inserts. Thus, it seems that the combination of two resistance mechanisms, NDM and PBP3 inserts, leads to a significant compromise in the cefepime/taniborbactam activity. This has been reported previously for isolates collected in China, wherein 28/29 NDM-expressing E. coli isolates with cefepime/taniborbactam MICs of >8 mg/L harbored amino acid insertions in PBP3 (31). In the present study, a significant cross-resistance between aztreonam/avibactam and cefepime/taniborbactam was also observed in E. coli, suggesting a deleterious impact of the amino acid insert in PBP3 on the activity of both these PBP3-targeting combinations. However, in the absence of NDM, unlike that on aztreonam/avibactam, the impact of PBP3 insert on cefepime/taniborbactam activity was relatively lower as against OXA-48-like-producing E. coli subset, 19/20 isolates were inhibited at ≤8 mg/L of cefepime/taniborbactam.

Among K. pneumoniae isolates, cefepime/taniborbactam MICs exceeded 8 mg/L for a substantial number of isolates producing NDM with or without OXA-48-like. In a previous study employing NDM-expressing K. pneumoniae isolates collected in the United States, the MIC_50_ and MIC_90_ of cefepime/taniborbactam were 1 and 32 mg/L, respectively (35), which is in agreement with our study. Since PBP3 inserts are not reported in K. pneumoniae, we tend to believe that the poor activity of cefepime/taniborbactam against such isolates could be linked with impermeability. This proposition is supported by another study involving K. pneumoniae, Enterobacter cloacae, and E. coli isolates wherein higher cefepime/taniborbactam MICs (≥4 mg/L) were linked to alterations in the major porins (29).

The pattern of cefepime/zidebactam activity against carbapenemase-producing Indian isolates observed in this study is in concordance with earlier investigations that tested U.S. and global isolates (20, 36–38). In these studies, the cefepime/zidebactam combination inhibited >99% of carbapenemase-expressing Indian isolates at ≤8 mg/L regardless of resistance mechanisms. Importantly, cefepime/zidebactam readily overcame the challenge of NDM with or without OXA-48-like plus PBP3 amino acid inserts in E. coli, which is attributed to zidebactam’s PBP2 binding-mediated β-lactam enhancer action (38). A concurrent multiple-PBP binding feature of cefepime/zidebactam is broadly analogous to that of carbapenems, which are also known to effectively overcome the PBP3 insert-based resistance mechanism, albeit only in isolates that do not produce carbapenemases (38). Such an advantage associated with multiple-PBP binding action is not available to cefepime/taniborbactam, aztreonam/avibactam, and ceftazidime-avibactam as they primarily target PBP3.

There was a single isolate with a cefepime/zidebactam MIC of > 8 mg/L. This was a K. pneumoniae isolate that harbored both NDM and OXA-48-like enzymes. The elevated MIC of cefepime/zidebactam against this isolate is possibly due to severe downregulation in the expression of outer membrane porins and/or hyperefflux in conjunction with NDM expression.

This study underscores that a β-lactamase-inhibitor-based approach may be intrinsically constrained in overcoming nonenzymatic resistance mechanisms such as PBP changes, efflux, and impermeability. Moreover, knowing the versatility of Gram-negative pathogens in evolving scores of β-lactamase variants, the β-lactam and β-lactamase-inhibitor approach would constantly face the challenge of newer β-lactamases.

In conclusion, the present study revealed the differential ability of cefepime/taniborbactam and cefepime/zidebactam in tackling carbapenemase-producing clinical isolates. While cefepime/taniborbactam activity was compromised against isolates producing NDM, cefepime/zidebactam retained a consistent activity regardless of the type of carbapenemase produced.

## MATERIALS AND METHODS

### Bacterial isolates.

Carbapenemase-expressing isolates used in this study (E. coli, *n* = 270, and K. pneumoniae, *n* = 300) were collected during 2019 to 2021 from nine Indian tertiary-care hospitals including Christian Medical College and Hospital, Tamil Nadu; Baby Memorial Hospital, Kerala; Dr. Somervell Memorial CSI Medical College, Kerala; Seth G.S. Medical College & KEM Hospital, Maharashtra; Panimalar Medical College, Hospital & Research Institute, Tamil Nadu; Meenakshi Medical College & Research Institute, Tamil Nadu; Pondicherry Institute of Medical Sciences, Pondicherry; Saveetha Medical College, Tamil Nadu; and Tata Medical Center, Kolkata. The isolates (nonduplicate, one isolate per patient) were retrieved from various clinical specimens including blood, urine, bronchial fluid, pus, catheter, rectal swabs, sputum, and stool. Bacterial species were identified using the matrix-assisted laser desorption ionization–time of flight (MALDI-TOF)-based Vitek mass spectrometer (MS) (bioMérieux, Marcy-l’Étoile, France). Carbapenemases were identified by PCR using previously described primers for *bla*_KPC_, *bla*_OXA-48-like_, *bla*_VIM_, *bla*_NDM_, and *bla*_IMP_ (39–41).

### MICs.

Cefepime, imipenem, meropenem, ceftazidime, and aztreonam were obtained from Sigma-Aldrich Company (St. Louis, MO, USA), and zidebactam, avibactam, relebactam, and vaborbactam were obtained from MedKoo Biosciences, Inc. (Morrisville, NC, USA). Taniborbactam was obtained from MolPort (Riga, Latvia). MICs were determined by the broth microdilution method following the M07 guidelines (11th edition) published by the Clinical and Laboratory Standards Institute (CLSI) (42). Cefepime/taniborbactam MICs were determined with a fixed 4-mg/L concentration of taniborbactam while cefepime/zidebactam MICs were determined at a 1:1 ratio as recommended by CLSI. Similarly, the CLSI-recommended concentration for β-lactamase inhibitors was used for other combinations: 4 mg/L for all, except 8 mg/L for vaborbactam (43).

### MAMA for PBP3 insert.

We developed sequencing-free mismatch amplification mutation assay (MAMA) PCR for the real-time detection of a 4-amino-acid insert (YRIK/YRIN) in PBP3 of E. coli isolates using forward (5′-CAACCCTAACAATCTGAG-3′) and reverse (5′-GTTAATTCGATAGTTAATTCGA-3′) primers. All carbapenemase-expressing E. coli isolates (*n* = 270) were screened for the presence of the PBP3 insert. The total volume of the PCR mixture was 20 μL with 5× master mix (Qiagen, Hilden, Germany), and the final concentration of the primers was 2 μM for the *ftsI* gene that codes for the PBP3 insert. Amplification was performed using the thermocycling condition of 95°C for 15 min for initial denaturation, 94°C for 30 s, 46°C for 1 min, and 72°C for 1 min for 30 cycles, and 72°C for 10 min. Amplified PCR products were run on 2% agarose and visualized on a Bio-Rad Gel Doc XR+ (Bio-Rad, Richmond, CA). This method was validated using whole-genome-sequenced appropriate positive-control (with PBP3 insert) and negative-control (without PBP3 insert) strains (see Table S1 in the supplemental material).
