# Supplementary material for: In Vitro Activity of Two Cefepime-Based Novel Combinations, Cefepime/Taniborbactam and Cefepime/Zidebactam, against Carbapenemase-Expressing Enterobacterales Collected in India
Source: Microbiol Spectr. 2023 Feb 27;11(2):e04925-22. doi: 10.1128/spectrum.04925-22 (PMC10100882; doi:10.1128/spectrum.04925-22)
Supplement: Supplemental file 1 — Table S1. Download spectrum.04925-22-s0001.docx, DOCX file, 0.02 MB [file spectrum.04925-22-s0001.docx]

Supplementary Table 1. List of *E. coli* strains used as positive and negative controls for validation of PCR based detection of four amino acid insert in PBP3 of *E. coli*

| **Positive control strains with 4 amino acid insertion in PBP3** | | | **Negative control strains (No amino acid insertion in PBP3)** | |
| --- | --- | --- | --- | --- |
| **Strain ID** | **Accession no.** | **4 amino acid insertion in PBP3** | **Strain ID** | **Accession no.** |
| BA22372 | CP040397.1 | YRIN | BA1067 | JACUWS000000000.1 |
| BP1473 | PVPT00000000.1 | YRIN | BA1166 | JACUWT000000000.1 |
| BV643 | PVPJ00000000.1 | YRIK | BA1289 | JACUWX000000000.1 |
| BA8153 | JACUYJ000000000.1 | YRIK | BA1314 | JACUWY000000000.1 |
| BP9671 | RCAC00000000.1 | YRIN | BA1320 | JACUWZ000000000.1 |
| FF2320 | JACUZE000000000.1 | YRIN | BA1341 | JACUXA000000000.1 |
| BA25291 | JACUXP000000000.1 | YRIK | BA1376 | JACUXB000000000.1 |
| BP3354 | PVPB00000000.1 | YRIK | BA155 | JACUXG000000000.1 |
| BA15282 | JACUXE000000000.1 | YRIN | BA177 | JACUXK000000000.1 |
| BA33057 | PVPV00000000.1 | YRIN | BA20856 | JACUXM000000000.1 |
| BA29338 | JACUXX000000000.1 | YRIK | BA24933 | JACUXO000000000.1 |
| BP6308 | JACUYV000000000.1 | YRIK | BA361 | JACUWR000000000.1 |
| B4814 | RCAM00000000.1 | YRIK | BA8689 | JACUYK000000000.1 |
| BA33222 | JACUYB000000000.1 | YRIK | BA924 | JACUYM000000000.1 |
| BA9666 | JACUYO000000000.1 | YRIK | BA929 | JACUYN000000000.1 |
| FF2501 | JACUZH000000000.1 | YRIK | FF1761 | JACUYZ000000000.1 |
| BA14872 | JACUXD000000000.1 | YRIN | FF1804 | JACUZA000000000.1 |
| BA12671 | JACUWW000000000.1 | YRIK | FF1971 | JACUZB000000000.1 |
| BA10937 | RCAG00000000.1 | YRIN | B7532 | SAZF00000000.1 |
| BA26754 | JACUXT000000000.1 | YRIN | B8110 | RCAL00000000.1 |
| BA7691 | JACUYI000000000.1 | YRIN | B8341 | SAZG00000000.1 |
| BA14242 | JACUXC000000000.1 | YRIK | B8538 | SAZH00000000. |
| BA25291 | JACUXQ000000000.1 | YRIK | B8571 | SAZI00000000.1 |
| BP14015 | JACUYT000000000.1 | YRIN | B8695 | SAZJ00000000.1 |
| BM1020 | JACUYP000000000.1 | YRIN | B9844 | SAZL00000000.1 |
| BA7390 | PVPM00000000.1 | YRIN | B9907 | SAZM00000000.1 |
| BA25715 | JACUXS000000000.1 | YRIK | BA6159 | RCAI00000000.1 |
| BP11998 | JACUYS000000000.1 | YRIN | BA9313 | PVPG00000000.1 |
| BA17743 | JACUXL000000000.1 | YRIN | BP1241 | RCAD00000000.1 |
| BA26818 | JACUXU000000000.1 | YRIK | BV723 | PVOT00000000.1 |
| BA28453 | JACUXW000000000.1 | YRIN |  |  |
| BA3848 | JACUYE000000000.1 | YRIN |  |  |
| FF1371 | JACUYY000000000.1 | YRIN |  |  |
| FF2156 | JACUZC000000000.1 | YRIN |  |  |
| FF2219 | JACUZD000000000.1 | YRIN |  |  |
| BA9615 | RCAH00000000.1 | YRIN |  |  |
| BA11691 | JACUWU000000000.1 | YRIN |  |  |
| BA16699 | JACUXI000000000.1 | YRIN |  |  |
| BA25514 | JACUXR000000000.1 | YRIN |  |  |
| BA29490 | JACUXY000000000.1 | YRIN |  |  |
| BA29686 | JACUXZ000000000.1 | YRIN |  |  |
| BA3696 | JACUYC000000000.1 | YRIN |  |  |
| BA8827 | JACUYL000000000.1 | YRIN |  |  |
| BP11051 | JACUYQ000000000.1 | YRIN |  |  |
| BP2876 | JACUYU000000000.1 | YRIN |  |  |
| BV313 | JACUYW000000000.1 | YRIN |  |  |
| FF2338 | JACUZF000000000.1 | YRIN |  |  |
| BA17642 | JACUXJ000000000.1 | YRIK |  |  |
| BP11855 | JACUYR000000000.1 | YRIN |  |  |
| BA1194 | JACUWV000000000.1 | YRIN |  |  |
| BA1529 | JACUXF000000000.1 | YRIN |  |  |
| BA15832 | JACUXH000000000.1 | YRIN |  |  |
| BA26837 | JACUXV000000000.1 | YRIN |  |  |
| BA505 | JACUYF000000000.1 | YRIN |  |  |
| BA29965 | PVOV00000000.1 | YRIN |  |  |
| BA7393 | JACUYH000000000.1 | YRIN |  |  |
| BA300 | JACUYA000000000.1 | YRIN |  |  |
| BA12315 | PVOW00000000.1 | YRIN |  |  |
| BA20936 | JACUXN000000000.1 | YRIK |  |  |
